# The Role of Granulocyte-Macrophage Colony-Stimulating Factor in Murine Models of Multiple Sclerosis

**DOI:** 10.3390/cells9030611

**Published:** 2020-03-04

**Authors:** Kelly L. Monaghan, Edwin C.K. Wan

**Affiliations:** 1 Department of Microbiology, Immunology, and Cell Biology, West Virginia University, Morgantown, WV 26506, USA; klm0031@mix.wvu.edu; 2 Department of Neuroscience, West Virginia University, Morgantown, WV 26506, USA; 3 Rockefeller Neuroscience Institute, West Virginia University, Morgantown, WV 26506, USA

**Keywords:** multiple sclerosis, experimental autoimmune encephalomyelitis, monocytes, granulocyte-macrophage colony-stimulating factor

## Abstract

Multiple sclerosis (MS) is an immune-mediated disease that predominantly impacts the central nervous system (CNS). Animal models have been used to elucidate the underpinnings of MS pathology. One of the most well-studied models of MS is experimental autoimmune encephalomyelitis (EAE). This model was utilized to demonstrate that the cytokine granulocyte-macrophage colony-stimulating factor (GM-CSF) plays a critical and non-redundant role in mediating EAE pathology, making it an ideal therapeutic target. In this review, we will first explore the role that GM-CSF plays in maintaining homeostasis. This is important to consider, because any therapeutics that target GM-CSF could potentially alter these regulatory processes. We will then focus on current findings related to the function of GM-CSF signaling in EAE pathology, including the cell types that produce and respond to GM-CSF and the role of GM-CSF in both acute and chronic EAE. We will then assess the role of GM-CSF in alternative models of MS and comment on how this informs the understanding of GM-CSF signaling in the various aspects of MS immunopathology. Finally, we will examine what is currently known about GM-CSF signaling in MS, and how this has promoted clinical trials that directly target GM-CSF.

## 1. Introduction

Multiple sclerosis (MS) is a chronic immune-mediated disease that impacts approximately 2.3 million people world-wide [1]. MS is characterized by the formation of demyelinating lesions, which are disseminated in both time and space. The location of the lesions correlates with the manifestation of physical disease symptoms [2]. In addition to demyelination, peripheral immune cell infiltration to the CNS is associated with inflammation, tissue damage, and axonal loss [3]. There are three major subtypes of MS: (1) relapsing remitting MS (RRMS), (2) secondary progressive MS (SPMS), and primary progressive MS (PPMS) [4,5]. RRMS is the most common subtype. This disease course is defined by periods of exacerbation followed by periods of clinical recovery, although new lesions can develop in clinically silent areas during periods of remission without the presentation of overt clinical symptoms [5]. A majority of RRMS patients will develop SPMS, which is defined as the progressive worsening of neurological dysfunction, without remission [5]. PPMS is less common and is defined as the accumulation of neurological dysfunction following onset of clinical symptoms with no remission [5]. While some studies have suggested that these three subtypes are one disease with differing clinical manifestations, it is important to distinguish between these subtypes. This is because the current disease-modifying agents that are used to treat MS are efficacious at treating neuroinflammation and abrogating some of the tissue damage and demyelination associated with the active phase of the disease, when patients exhibit overt clinical symptoms [6,7,8]. However, these same disease-modifying agents are not efficacious at impeding disease progression [6,7,8]. Consequently, the major focus in the field of MS research is to develop novel therapeutic strategies to dampen neuroinflammation and prevent MS progression.

Animal model systems of MS have provided insight into the immunopathology of MS. Studies in these models have directly and indirectly contributed to the development of disease-modifying agents that are utilized in the clinic [8]. The most widely studied murine model of multiple sclerosis is experimental autoimmune encephalomyelitis (EAE). This animal model closely recapitulates the neuroinflammatory process that is associated with MS [9]. Consequently, this model has been used to identify novel therapeutic targets by ascertaining those mediators that are critical for potentiating neuroinflammation. One such mediator that has gained attention for its role in promoting EAE-associated inflammation is the cytokine granulocyte-macrophage colony-stimulating factor (GM-CSF). This cytokine first drew attention when a clinical report in 1998, which assessed cytokine concentrations in the cerebral spinal fluid of MS patients with active disease, found that the levels of GM-CSF are significantly increased in MS patients compared to healthy controls [10]. Based on this observation, McQualter and colleagues wanted to determine whether GM-CSF played a critical and non-redundant role in promoting EAE pathology. [11]. This study, which will be discussed in detail later in this review, is the first to underscore the critical role of GM-CSF in potentiating EAE pathology. Based on their findings and the aforementioned clinical study, McQualter and colleagues posited that GM-CSF is a putative therapeutic target for MS treatment [11]. Since then, much information has been gleaned about the role of GM-CSF in EAE pathology, including the cells types that produce and respond to this cytokine. It is evident from recent studies that GM-CSF plays a dynamic role in mediating EAE pathology. In this review, we will explore the current findings related to the function of GM-CSF signaling in EAE pathology. We will then assess the role of GM-CSF in alternative models of MS and comment on how this informs the understanding of GM-CSF signaling in the various aspects of MS immunopathology. Finally, we will explore the studies that have directly ascertained the function of GM-CSF in MS, and what implications these findings have for developing novel therapies that target GM-CSF and its downstream mediators.

## 2. GM-CSF

### 2.1. Protein Structure, Receptor Structure, and Signaling

GM-CSF is a 114 amino acid polypeptide that is secreted as a monomeric 23kDA glycosylated small glycoprotein protein, though the molecular weight can vary depending on the extent of glycosylation [12]. Human *CSF2* is encoded by 2.5kb mRNA that consists of four exons on the chromosome region 5q31 [12,13]. Murine and human GM-CSF share 70% nucleotide and 56% sequence homolog, suggesting that while cross-reactivity between human and murine GM-CSF does not occur, murine models can be utilized to study the role of GM-CSF in the context of human diseases [12]. The GM-CSF receptor is a heterodimer that consists of an α subunit and a common beta chain (βc) subunit, which is shared with IL-3 and IL-5 [14]. Interestingly, functional mutagenesis studies and crystal structure analysis of the GM-CSF receptor demonstrate that receptor activation is predicated on the assembly of the GM-CSF receptor into a dodecamer or higher order structure [15]. Activation of the GM-CSF receptor requires both the α subunit and βc subunit. The βc subunit is associated with Janus kinase 2 (JAK2); however, the βc subunit keeps its tails far enough apart that transphosphorylation of JAK2 cannot occur [16,17]. When GM-CSF binds to the receptor, the higher order dodecamer complex brings the subunit tails close enough together to mediate the interaction between the JAK2 molecules, resulting in functional dimerization and transphosphorylation [15,17]. The activation of JAK2 results in the activation of the signal transducer and activator of transcription 5 (STAT5). STAT5 can then translocate to the nucleus and regulate the expression of target genes [18]. GM-CSF is known to play an indispensable role of JAK2-STAT5 signaling [19]. GM-CSF can also activate the interferon regulatory factor 4 (IRF4)-CCL17 pathway which is associated with pain [20]. GM-CSF signaling activates IRF4 by enhancing the activity of JMJD3 demethylase [20]. The upregulation of IRF4 results in an increased expression of MHC II by differentiating monocytes and an increase in the production of CCL17 [20]. Additionally, GM-CSF signaling is implicated in the AKT-ERK mediated activation of NF-κB [21]. Given the pleiotropic nature of GM-CSF, it is unsurprising that this cytokine plays a major role in both maintaining homeostasis and promoting inflammation.

### 2.2. Cellular Source and Function of GM-CSF during Homeostasis

GM-CSF is a pleiotropic cytokine that is known to be a major mediator in inflammation; however, GM-CSF also functions in maintaining homeostasis. In the lungs, GM-CSF is abundantly produced by epithelial cells. Murine studies utilizing GM-CSF-deficient mice (*Csf^−/−^*) reveal that GM-CSF is required for the development of functional alveolar macrophages through the regulation of the transcription factor PU.1 [22,23]. Given that alveolar macrophages play a major role in facilitating the clearance of surfactant from the alveolar space, GM-CSF-deficient mice develop a condition known as pulmonary alveolar proteinosis (PAP), which is characterized by the accumulation of surfactant in the alveolar space [23,24]. Further investigation posited that GM-CSF signaling directly regulates the differentiation of liver-derived fetal monocytes into immature alveolar macrophages during embryonic development [23]. GM-CSF signaling also promotes the differentiation of immature alveolar macrophages into mature alveolar macrophages, postnatally [23]. Intriguingly, immunocompromised patients that develop cryptococcal meningitis have circulating anti-GM-CSF autoantibodies. These patients exhibit reduced surfactant clearance, and a number of these patients subsequently developed PAP [25].

In addition to promoting the development of alveolar macrophages, GM-CSF also appears to play a minor role in the development of tissue-resident conventional dendritic cells (cDCs). *Csf2^−/−^* or *Csfr2^−/−^* mice have fewer CD103+ cDCs in the lung, dermis, and intestine [24,26,27]. In other lymphoid tissues, however, tissue-resident cDC development appears to be normal [28]. This is an interesting observation given that, under inflammatory conditions, GM-CSF is a major cytokine that promotes monocyte differentiation into dendritic cells, and a more critical role of this cytokine in cDC development is anticipated [29]. Since GM-CSF and its downstream mediators are potential therapeutic targets, it is necessary to consider the role that GM-CSF plays in the development of both alveolar macrophages and cDCs to prevent undesirable and potentially dangerous off-target effects.

### 2.3. GM-CSF in Murine Models of Multiple Sclerosis

#### GM-CSF in Experimental Autoimmune Encephalomyelitis

Experimental autoimmune encephalomyelitis (EAE) is the most well-studied model of multiple sclerosis. This model was established in 1933 by Rivers and colleagues in an attempt to address human encephalitis resulting from rabbit spinal cord contamination in the human rabies vaccine [30]. Since its development, rodent and primate models have utilized some variation of this model to generate acute monophasic, relapsing–remitting, and chronic inflammatory phenotypes [31]. Given that the role of GM-CSF has been elucidated in murine EAE models, we will focus on murine models for the remainder of this review. EAE can be induced through two mechanisms [32]. The first is active EAE induction, whereby myelin or brain tissue peptides such as myelin oligodendrocyte glycoprotein amino acid 35-55 (MOG_(35–55)_), myelin basic protein (MBP), or proteolipid protein (PLP) are emulsified in complete Freund’s adjuvant (CFA) and subcutaneously injected into naïve recipient mice [33]. This is followed by two intraperitoneal injections (IP) of pertussis toxin at 2- and 48-h post induction. The pertussis toxin is thought to increase the permeability of the blood–brain barrier, thereby facilitating peripheral immune cell infiltration into the CNS parenchyma [34]. The resulting clinical presentation of active EAE induction is contingent on the strain of mice being utilized. For example, when EAE is induced via active induction with MOG _(35–55)_ in CFA in mice on a C57BL/6J background, the mice develop a monophasic and chronic disease pattern that is characterized by white matter demyelination and peripheral CD4+ T cell and myeloid cell infiltration [35]. The onset of clinical symptoms usually appears between days 9–10, and the symptoms reach peak severity between days 13–15 [35]. Active EAE induction in C57BL/6 mice is a valuable tool for recapitulating the immune cell infiltration and resulting neuroinflammation that mediate MS pathology [31]. In addition, EAE is commonly induced in SJL/J mice using PLP_(139–151)_. Active EAE induction in the SJL/J mice results in a relapsing–remitting disease course which is characterized by peripheral immune cell infiltration, inflammation, and demyelination (relapses), followed by the resolution of inflammation but the progression of white matter damage and axonal damage with no overt clinical symptoms (remission) [31]. This model is a useful tool to study relapsing–remitting MS [32]. The other major mechanism to induce EAE is through the adoptive transfer of pathogenic CD4+ T cells. In this model, antigen-specific CD4+ T cells are transferred to naïve recipient mice to induce EAE. In this model, the priming phase of EAE that occurs in the periphery is bypassed, therefore the in vitro manipulation of CD4+ T cells prior to transfer can allow researchers to study the role of various cytokines during the effector phase of EAE [33]. Neither active nor passive EAE induction completely recapitulates all aspects of MS immunopathology; however, EAE is a useful tool to study various aspects of the immune-mediate response. This is evidenced by the successful development of standard-of-care MS disease-modifying agents utilizing EAE models, including interferon beta, glatiramer acetate, and natalizumab (anti-alpha 4 beta 1 integrin) [36,37,38]. Though the exact mechanism has not been fully elucidated, interferon beta is thought to act as an immunomodulatory agent that dampens inflammation in the CNS [39]. Additionally, interferon beta is also thought to prevent the migration of proinflammatory immune cells into the CNS [39]. Glatiramer acetate is a synthetic amino acid copolymer that is thought to expand the regulatory T cell population in the periphery, which can migrate into the CNS parenchyma and produce anti-inflammatory mediators that abrogate the activation of immune cells that are reactive against myelin [40]. Natalizumab binds to the α_4_ subunit of α_4_β_1_ integrin on the surface of lymphocytes, which prevents binding to the vascular cell adhesion molecule 1 (VCAM-1). This prevents T cells from migrating into the CNS parenchyma [41,42]. Consequently, EAE is currently the best model to understand the role of GM-CSF in MS pathogenesis and its therapeutic implications.

**Figure 1:**
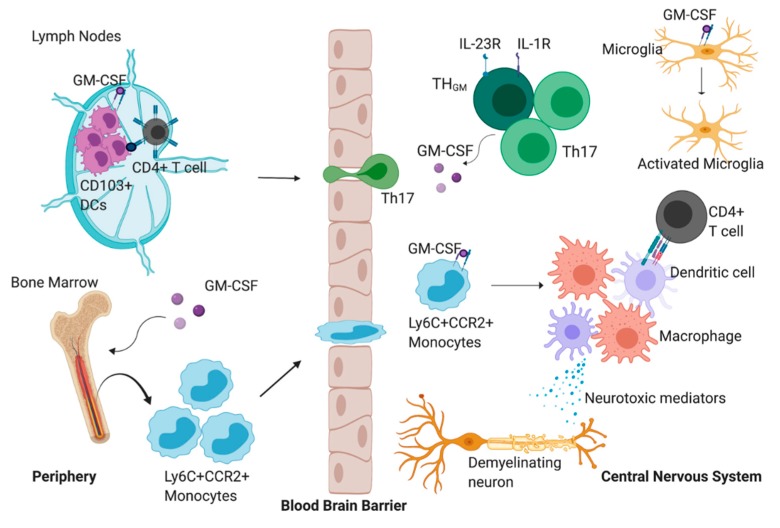
The proposed role of granulocyte-macrophage colony-stimulating factor (GM-CSF) during experimental autoimmune encephalomyelitis (EAE). GM-CSF promotes the accumulation of CD103+ dendritic cells (DCs) in the lymph nodes which can present myelin antigen to CD4+ T cells. These CD4+ T cells can then migrate into the central nervous system (CNS) parenchyma where they begin to produce GM-CSF exclusively, or GM-CSF and IL-17. GM-CSF production by the CD4+ T cells promotes the migration of Ly6C+CCR2+ cells from the bone marrow to the CNS. Once in the CNS, GM-CSF signaling promotes the differentiation of monocytes into a heterogenous population of monocyte-derived macrophages and monocyte derived dendritic cells. Monocyte-derived dendritic cells can interact with and promote the activation of infiltrating CD4+ T cells. In addition, these differentiated cells can secrete mediators that directly promote demyelination, tissue damage and axonal loss. GM-CSF can also promote the activation of CNS-resident microglia. These reactive microglia can potentiate the inflammatory milieu by producing proinflammatory mediators.

The first study to assess the role of GM-CSF in EAE pathology was conducted by McQualter and colleagues in 2001. Their goal was to determine whether GM-CSF played a critical and non-redundant role in promoting EAE pathology, which was based on previous findings suggesting that that concentration of GM-CSF was increased in the cerebral spinal fluid of patients with MS compared to healthy controls [10]. To this end, they generated a GM-CSF-deficient mouse that was backcrossed to an EAE-suspectable NOD/Lt background. EAE was induced through active induction with MOG _(35–55)_ and the clinical presentation in this particular stain of mice was a relapsing–remitting biphasic phenotype. The study found that, although functionally normal in terms of hematopoiesis, these mice are resistant to the EAE, which was demonstrated by the lack of immune cell infiltration into the CNS in addition to the absence of clinical symptoms, suggesting that GM-CSF is important for the development of demyelinating lesions and the migration and/or expansion of immune cells within the CNS [11]. These findings suggest that GM-CSF is a conceivable threptic target for MS. In order to develop these novel therapies, it is necessary to understand the cell types and subsequent signaling pathways that regulate the production and response to GM-CSF. The proposed role of GM-CSF during EAE is detailed in Figure 1.

### 2.4. T cells Are the Predominant Source of GM-CSF during EAE

In 2007, a study published by Ponomarev and colleagues identified the cellular source of GM-CSF during EAE as T cells and not CNS-resident microglia or other infiltrated peripheral immune cells [43]. This study suggests that Th1 CD4+ T cells are the major T cell subset that produce GM-CSF. This idea that Th1 cells are the major source of inflammation in EAE is due to the fact that both IL-12 and IL-23 share the p40 subunit, therefore any efficacious strategies that blocked IL-12p40 subsequently block both IL-12 and IL-23 activity [44]. With the discovery of IL-23 and Th17 cells, however, the notion that Th1 cells are the predominant source of inflammation during EAE was quickly challenged [45,46]. One study that challenges this notion demonstrates that while it is true that the passive transfer of IL-12p70- and IL-23-polarized cells can cause EAE, treatment with anti–GM-CSF can ameliorate EAE induced in the mice that receive IL-23 polarized Th17 cells, but not IL-12p70 polarized Th1 cells [47]. This suggests that Th17, but not Th1 cells, are the major source of GM-CSF during EAE. The role of Th17 cells in EAE is further supported by an elegant study that demonstrated that the upregulation of both IL-23 and RORγt license the Th17 cells to produce GM-CSF. IL-12 and IFNγ on the other hand, are negative regulators of GM-CSF production by these cells [48]. Furthermore, GM-CSF secretion from *Ifng*^−/−^*Il17a*^−/−^ mice was sufficient to induce EAE; however, *Csf2^−/−^* mice, which lack GM-CSF, do no develop EAE, suggesting that other inflammatory mediators are not sufficient to induce pathology [48]. A study by Mangalam and colleagues found that IFNγ sequesters infiltrating immune cells to the spinal cord during EAE, and it partially suppresses the production of GM-CSF by Th17 cells, rendering them less pathogenic [49]. Additional studies demonstrate that targeting other Th17-associated cytokines including IL-17F, IL-22, and IL-21 does not confer resistance to EAE [50,51,52]. These findings indicate that GM-CSF is the major Th17-associated cytokine that licenses the CD4+ T cells to become encephalitogenic. In fact, GM-CSF is now thought to be the only Th17-associated cytokine that had a non-redundant function in promoting EAE pathology [53]. A recent study found that GM-CSF production by Th17 cells is not restricted to upstream regulation by IL-23. This study showed that STAT5 deficiency in CD4+ T cells confers resistance to EAE by impairing the expression of GM-CSF [54]. Further investigation found that IL-7 acts upstream of STAT5. The study posited that the CD4+ T cells regulated by the IL-7-STAT5 axis are a distinct subset of Th cells, which they named Th_GM_ cells. These cells have minimal expression of the master gene regulators of Th1 and Th17 cells, T-bet and RORγt. Microarray analysis revealed that the Th_GM_ cells co-express GM-CSF and IL-3, which is not the case in either Th1 or Th17 cells. Additionally, when the three Th subtypes were adoptively transferred into *Rag2^-/-^* mice, the Th_GM_ cells caused more severe EAE compared to EAE induced by the transfer of Th1 or Th17 cells [54]. A recent study further supported the notion that Th_GM_ are a distinct subset of pathogenic Th cells. To this end, they generated a fate-map and reporter of GM-CSF expression mouse stain, whereby they were able to identify a subset of Th cells that required IL-23R and IL-1R signaling but not IL-6R signaling, to promote pathogenesis [55]. Furthermore, when this subset of Th cells was ablated, the inflammatory cascade was perturbed; however, the accumulation of Th1 and Th17 cells were not impacted, further underscoring the notion that these cells are a distinct subset of GM-CSF Th cells [55]. Interestingly, the production of GM-CSF may be dependent on a subset of CCR4-expressing dendritic cells [56]. When CCR4 expression was ablated in this cell subset, these cells showed a significant decrease in the expression of IL-23 [56]. Consequently, these mice were protected against EAE and had less GM-CSF overall in the spinal cords, suggesting that CCR4 expression on DCs maintains the Th17 population, thereby regulating the production of GM-CSF [56]. It is evident from the aforementioned findings that CD4+ T cells are the major cellular source of GM-CSF during EAE. Additional studies are required to confirm that Th_GM_ cells are, in fact, a distinct subset of Th cells during EAE. These cells may serve as novel therapeutic targets. A list of cell types that produce GM-CSF during EAE is summarized in Table 1.

### 2.5. Many Immune Cells Respond to GM-CSF during EAE

Once CD4+ T cells were found to be the major cellular source of GM-CSF during EAE, there was a need to understand which immune cell types were responding to the high level of GM-CSF that was being produced by these cells. One of the first studies to address this question suggested that myeloid cells are a major component of the inflammatory infiltrate, and these cells must migrate into the CNS prior to EAE relapses [70]. Using GM-CS-deficient mice, King and colleagues demonstrated that GM-CSF promotes CD11b^hi^ Ly6C^hi^ egress from the bone marrow, across the blood–brain barrier, and into the CNS parenchyma, where these cells will upregulate the expression of proinflammatory cytokines [70]. Additional studies demonstrate that GM-CSF deletion results in fewer monocyte-derived cells in the CNS parenchyma following EAE induction, and the overexpression of GM-CSF results in increased monocyte migration, which underscores the role of GM-CSF in mediating monocyte migration from the bone marrow into the CNS parenchyma [11,71,72]. The conditional deletion of the *Csf2r* on various immune cells, including CD103+ conventional dendritic cells, CNS-resident microglia, and neutrophils, does not alter the progression of EAE [64]. However, when *Csf2r* is deleted on CCR2+Ly6C+ monocytes, the mice are resistant to EAE and have a similar phenotype to the complete *Csf2 ^−/−^* mice, suggesting that this particular subset of monocytes responds to GM-CSF and is critical in mediating EAE pathology [64]. It is thought that, in addition to promoting the migration of CCR2+Ly6C+ cells into the CNS, GM-CSF is required to promote the differentiation of these specific infiltrated monocytes into antigen-presenting cells which can subsequently produce proinflammatory cytokines and present antigen to and maintain the pathogenic CD4+ T cell population [62,73]. In fact, Helft and colleagues found that when bone marrow monocytes are treated with GM-CSF in vitro, the resulting population is heterogenous in nature, and is comprised of monocyte-derived dendritic cells and macrophages, supporting the idea that GM-CSF promotes monocyte differentiation [29]. There is also evidence to suggest that, once in the parenchyma, the monocyte-derived cells can produce mediators that directly promote tissue damage, demyelination, and axonal loss [63,65].

In addition to monocytes, there are other cell-types that can respond to GM-CSF during EAE. The accumulation of CD103+ dendric cells in the lymph nodes is dependent on the presence of GM-CSF [66]. The CD103+ dendric cells present myelin antigen to, and subsequently activate, naïve CD4+ T cells, and therefore contribute to the onset of EAE [66]. However, when the *Csf2r* is conditionally deleted in CD103+ dendritic cells, severe EAE could still be observed in these mice, suggesting that *Csf2r* expression on the CD103+ dendric cells is not exclusively required for EAE initiation and/or progression [64]. Neutrophils are an additional myeloid cell type that is known to respond to GM-CSF [74]. Studies using anti-CXCR2, a major chemoattractant for neutrophils, demonstrate that inhibiting the activity of this chemokine confers protection against EAE [75]. Furthermore, GM-CSF is thought to promote the accumulation of neutrophils in the brain of mice with atypical EAE, wherein mice exhibit extensive inflammation in both the brain and spinal cord [67]. Therefore, neutrophils may be an important cell type that respond to GM-CSF and subsequently promote EAE pathology. In addition to myeloid cells, CNS-resident microglia become activated in response to GM-CSF produced by infiltrating CD4+ T cells prior to the onset of clinical symptoms, suggesting that GM-CSF-dependent microglial activation is required for the progression of EAE [43,68]. However, there have been very few studies to assess how GM-CSF promotes the activation of microglia during EAE. This will require further investigation. In addition, astrocytes are known to promote EAE pathology [76]. In a recent study, Wheeler and colleagues utilized transcriptome analyses to characterize astrocyte activation during EAE in response to GM-CSF signaling [69]. They found that GM-CSF stimulation promoted the expression of MAFG and MAT2α which are thought to repress anti-inflammatory transcriptional programs. In addition, GM-CSF stimulation in astrocytes promoted proinflammatory transcriptional programs, suggesting that GM-CSF signaling in astrocytes renders them pathogenic in the context of EAE [69]. It is evident that monocytes are the predominant cell type that respond to GM-CSF during EAE. Consequently, targeting GM-CSF and/or the downstream mediators of GM-CSF signaling in these cells may be a promising therapeutic approach to curtail pathogenic monocyte infiltration and differentiation in the CNS. A list of cell types that respond to GM-CSF during EAE is summarized in Table 1.

### 2.6. GM-CSF in Other Murine Models of MS

The role of GM-CSF in less commonly used models of MS has not been well elucidated. One model that may depend on GM-CSF signaling is the Theiler’s murine encephalomyelitis virus-induced demyelinating disease (TMEV-IDD) [77]. Theiler’s murine virus is an enteric commensal in most mouse stains; however, when injected via intracranial injection into susceptible mice, such as SJL/J mice, the result is a chronic and progressive demyelinating disease [78,79]. The chronic phase of TMEV-IDD is characterized by inflammation, demyelination, axonal degeneration, and astrogliosis, making this a suitable model to study MS progression [79]. One study suggested that GM-CSF may play a role in promoting pathology in this model. Bone marrow cells stimulated with GM-CSF were infected with TMEV, and the presence of GM-CSF was found to promote virus replication and the production of proinflammatory cytokines, indicating that GM-CSF is important in inducing TMEV-IDD [77]. The importance of GM-CSF in this model further highlights the important role of GM-CSF in promoting neuroinflammation associated with immune cell infiltration into the CNS parenchyma, although additional in vivo studies need to be performed to further characterize the role of GM-CSF in this model of demyelinating disease. Interestingly, in the Cuprizone model, which is a non-inflammatory model of MS that promotes demyelination by promoting the death of mature myelin-producing oligodendrocytes, there is no literature to support the notion that GM-CSF plays a role in promoting pathology [80,81]. This suggests that GM-CSF does not directly facilitate demyelination, rather it promotes the differentiation and activation of immune cells that can then directly promote demyelination. Consequently, as therapies are being developed, co-treatment with a mediator that prevents demyelination by protecting mature oligodendrocytes should be considered.

### 2.7. Controversy over GM-CSF in Murine Models of MS

Studies from animal models have convincingly demonstrated that GM-CSF plays a critical role in promoting EAE pathology. However, recent studies have brought the importance of this cytokine in the onset of disease into question. The first study performed by Pierson and Goverman sought to determine the role of GM-CSF in EAE that is induced in C3HeB/FeJ mice, which develop an inflammatory disease in both the brain and spinal cord [67]. This model is unique because the inflammation resulting from EAE induced in mice on a C57BL/6 background has a strong predilection for the spinal cord [31]. GM-CSF-deficient C3HeB/FeJ mice develop EAE because IL-17 is able to compensate for the loss of GM-CSF, and is able to promote neutrophil accumulation, inflammation, and demyelination [67]. Interestingly, this study also determined that the co-expression of IL-17 and GM-CSF is required to promote immune cell migration into the brain, which is normally inhibited by IFNγ [79]. This suggests that, while GM-CSF may be important for promoting EAE pathology, it is not the only cytokine that is essential. Therefore, when therapeutics are being generated, an approach that involves targeting multiple cytokines, including GM-CSF, should be considered. This notion is further supported by a study that posited that GM-CSF is required for the accumulation of pathogenic CD4+ T cells in the lymph nodes, but is not required for the activation of these cells, and is therefore not required for the onset of EAE [71]. To determine whether or not this was the case, active EAE was induced in *Csf2^−/−^* mice, and the number of pathogenic CD4+ T cells in the lymph nodes was significantly decreased, suggesting that the accumulation of CD4+ T cells in the lymph nodes is dependent on GM-CSF [71]. However, when these T cells were expanded in vitro under Th17-polarizing conditions, and were adoptively transferred to wild-type recipient mice, these cells were able to induce EAE, suggesting that GM-CSF is not exclusively required for the development of encephalitogenic CD4+ T cells [71]. Additionally, when wild-type pathogenic CD4+ T cells were adoptively transferred to *Csf2^−/−^* mice, the onset of clinical EAE symptoms was unaltered; however, perturbing GM-CSF signaling did alter the immune cell profile in the CNS, thereby decreasing disease severity and preventing the progression to chronic disability [71]. This study suggests that GM-CSF is indispensable for promoting EAE onset; however, GM-CSF signaling is compulsory for EAE progression. These findings emphasize the importance of considering the role of additional cytokines that can be therapeutically targeted in conjunction with GM-CSF.

## 3. GM-CSF in MS

### 3.1. Immune Cells that Produce and Respond to GM-CSF during MS

Animal models of MS have allowed us to gain significant insight into the role of GM-CSF in promoting this immune-mediated disease. Despite the breakthroughs in our understanding of this cytokines in murine models, the role of GM-CSF in MS is still not completely elucidated. It has been known for some time that the concentration of GM-CSF is significantly increased in the cerebral spinal fluid (CSF) of patients with active MS compared to healthy controls [10]. Similar to murine EAE models, GM-CSF is thought to be produced by CD4+ T cells that contain an MS-associated polymorphism in the IL-2 receptor alpha gene [82]. Similar to EAE, a distinct subset of CCR6-expressing Th cells that exclusively produce GM-CSF have been found in high numbers in the CSF of patients with active disease, suggesting that CD4+ T cells are the major cellular source of GM-CSF during MS [83]. In fact, efficacious treatment with the disease-modifying agent interferon beta significantly decreases the number of GM-CSF-producing CD4+ T cells in the peripheral blood and in the CSF of patients with MS compared to untreated patients [59]. In addition to CD4+ T cells, GM-CSF is produced by a subset of B cells and CD8+ T cells during active MS [59,60]. The recent success of the disease-modifying agent Ocrelizumab, which depletes CD20-expressing B cells, for the treatment of progressive MS, suggests that pathogenic B cells may play an important role in mediating MS pathology [84]. Additional mechanistic studies are needed to address the role of GM-CSF-producing B and T cells in MS. Such mechanistic studies can inform more efficacious therapeutics to prevent MS progression.

Similar to EAE, monocytes appear to be the major cell types that respond to GM-CSF in MS [85]. GM-CSF increases the migration of monocytes across the blood–brain barrier and, once in the parenchyma, promotes the differentiation of monocytes into monocyte-derived antigen-presenting cells [85]. Analyses of postmortem brain tissue obtained from patients with MS demonstrate that these monocyte-derived antigen-presenting cells are the predominant cell type found at the site of active demyelinating lesions [85,86,87,88]. Moreover, these same cells have been found to persist at the sites of chronic demyelinating lesions [89]. Since GM-CSF plays a critical role in promoting the migration of these cells across the blood–brain barrier, targeting GM-CSF therapeutically may prevent the migration of pathogenic monocyte-derived cells into the CNS. Additionally, an analysis of active and chronic demyelinating lesions found that the expression of the GM-CSF receptor is highly upregulated on the lesion-associated astrocytes and microglia, suggesting that both of these CNS-resident cells may upregulate proinflammatory genes in response to GM-CSF signaling [90]. Additional studies are required to ascertain the role of GM-CSF signaling in these CNS-resident cells, and how this signaling contributes to MS pathology.

### 3.2. Clinical Trials Therapeutically Targeting GM-CSF

There are numerous clinical trials that are attempting to target GM-CSF or the GM-CSF receptor for the treatment of autoimmune diseases [91]. One biologic that has been tested in clinical trials as an MS therapy is MOR103 [91]. MOR103 is a humanized monoclonal antibody against GM-CSF [92]. In a 20-week, randomized, double-blind, placebo-controlled phase 1b dose-escalation trial, patients with relapsing–remitting MS or secondary progressive MS with less than 10 gadolinium-enhancing lesions were administered through intravenous infusions of either MOR103 or a placebo control [92]. The primary objective of this study was safety, and although MOR103 demonstrated only modest efficacy, it was well-tolerated in patients with MS, and overall had a favorable safety profile [92]. This study is important, because there are some risks associated with blocking the biological activity of GM-CSF, which are important to keep in mind when developing a therapy against GM-CSF. Targeting GM-CSF and its receptor has been associated with exacerbations in pre-existing intestinal inflammation and the onset of colitis [93,94]. Additionally, as was previously mentioned, GM-CSF signaling is critical for the development of alveolar macrophages [22,23]. The accumulation of autoantibodies against GM-CSF is associated with an increased risk of developing pulmonary alveolar proteinosis, which is characterized by decreases in alveolar macrophages which result in the abnormal accumulation of surfactant in the lungs [95]. Additional clinical trials will need to further evaluate the efficacy of MOR103. Although this is the only clinical trial that has assessed the use of anti-GM-CSF or GM-CSF receptor inhibitors to treat MS, there are numerous clinical trials that are attempting to utilize these biologics to treat Rheumatoid Arthritis [91]. Many of these therapeutics will likely also be tested for efficacy to treat MS in the future.

## 4. Conclusions

Murine models of MS have allowed us to gain insight into the important role that GM-CSF plays in mediating neuroinflammation. These models have proven to be a useful tool for studying immune cell infiltration and the resulting inflammatory milieu, given that many of the major underpinnings of EAE pathology have been validated in patients with MS. These models have established that CD4+ T cells are the major cellular source of GM-CSF during EAE, and the monocytes are the major cell type that responds to that GM-CSF. These monocytes can then infiltrate into the CNS and promote inflammation, demyelination, and axonal loss. These cells and their downstream mediators are ideal targets for MS therapies. However, there is still much to be learned about the role of GM-CSF in mediating EAE pathology. Additional studies need to access the importance of Th_GM_ cells, the role of GM-CSF signaling in lesion-associated microglia and astrocytes, and the functional importance of GM-CSF-producing B cells in patients with MS. Despite these shortcomings in the literature, GM-CSF appears to be a promising therapeutic target for treating MS progression. Evidence from the literature also strongly alludes to the notion that a combined therapy approach that includes inhibiting the biological function of GM-CSF will likely be the most efficacious approach to treat MS.

## Figures and Tables

**Table 1:** Immune cell types that produce or respond to GM-CSF during EAE.

**Cell Types that Produce GM-CSF**	**Cellular and Molecular Signals Involved**
Th17 cells	IL-23-mediated expression of RORγt [48,57].
Th_GM_	IL-7-mediated activation of STAT5 [54]; IL-23R and IL-1R signaling [55].
CD8+ T cells	IL-23 induces but IFN-β suppresses GM-CSF production [58,59].
B cells	B-cell receptor, CD40, and IL-4-mediated STAT5/6 activation [60].
Dendritic cells	CCL17/CCL22-mediated expression of GM-CSF via CCR4 [56].
CNS endothelial cells	Monocyte-produced, IL-1β-mediated expression of GM-CSF [61].
**Cell Types that Respond to GM-CSF**	**Cell Type-Specific Biological Function of GM-CSF during EAE**
Monocytes	Stimulates CNS migration; induces the production of proinflammatory cytokines and neurotoxic mediators; promotes cell differentiation [62,63,64,65].
Dendritic cells	Induces the production of IL-23 that promotes EAE [56].
CD103+ dendritic cells	Induces cell accumulation in the skin and peripheral lymph nodes that can then present antigen to pathogenetic CD4+ T cells [66].
Neutrophils	Promotes cell accumulation in the brain that causes atypical EAE [67].
Microglia	Induces activation and promotes onset of EAE [43,68].
Astrocytes	Promotes the upregulation of proinflammatory gene expression [69].
